# U.S. Undergraduate Education in Public Health: Hot or Not?

**DOI:** 10.3389/fpubh.2015.00071

**Published:** 2015-05-11

**Authors:** Yelena N. Tarasenko, Joel M. Lee

**Affiliations:** ^1^Department of Health Policy and Management, Jiann-Ping Hsu College of Public Health, Georgia Southern University, Statesboro, GA, USA; ^2^Department of Epidemiology, Jiann-Ping Hsu College of Public Health, Georgia Southern University, Statesboro, GA, USA; ^3^Department of Health Policy and Management, College of Public Health, The University of Georgia Health Sciences Campus, Athens, GA, USA

**Keywords:** undergraduate public health education, baccalaureate degree in public health, baccalaureate program in public health, undergraduate students, college directories, websites

## Abstract

**Objectives:**

Undergraduate public health education has received growing attention in recent years. This includes a *Washington Post* article referring to undergraduate public health education as a “hot field” for a global generation, the Critical Component Elements of an Undergraduate Major in Public Health developed by the Association of School and Programs in Public Health (ASPPH), and a recent report from the de Beaumont Foundation and ASPPH. To evaluate the demand for the degree and assess the current state of undergraduate public health education, the researchers examined the number and characteristics of publicly reported U.S. baccalaureate public health programs.

**Methods:**

The researchers reviewed three 2013 college directories and the ASPPH website and identified 112 institutions that used the term “public health” in their baccalaureate degree listings that guide prospective students in selecting an academic program. The researchers defined the undergraduate degree in public health as a major leading to a B.S., B.A., or other baccalaureate degree in public health or public health studies that provides students with a strong general background in areas of knowledge basic to public health, or a specialized training in at least one of the five core disciplines of public health. The researchers then compared the degree contents as listed in the directories to the institutions’ websites to verify offering a public health curriculum. Carnegie Commission on Higher Education’s classifications of colleges and universities were applied to assess the characteristics of institutions offering baccalaureate degrees in public health.

**Results:**

Only 54 of the 2,968 U.S. institutions of higher education provided online information meeting the definition of an active undergraduate public health degree program.

**Conclusion:**

While public health may be a “hot” field in terms of the interest that it generates, the actual number of verified undergraduate programs presently available is relatively modest.

A 2008 *Washington Post* article received a great deal of national attention when it referred to public health as a “hot field” for a global generation (1). The article noted that courses in epidemiology, public health, and global health had been “drawing undergraduates to lecture halls in record numbers, prompting a scramble by colleges to hire faculty and import ready-made courses,” and “schools that have taught the subjects for years have expanded their offerings in response to surging demand” (1).

A recent report documents an increase in the number of public health degree conferrals from 759 in 1992 to 1,469 in 2004 and 6,464 by 2012 (2). In an effort to “assure conditions in which people can be healthy” (3), the Institute of Medicine (IOM) recommended that all undergraduates should have access to public health education. The intent was to prepare an “educated citizenry” as well as a well-educated public health workforce (4). The recommendation sparked the Educated Citizen and Public Health Movement (5–7). The movement encourages undergraduate public health core curricula as part of general education at 4-year and 2-year colleges, as well as integration of public health throughout undergraduate education (5–8).

Estimates of the number of institutions awarding undergraduate public health degrees vary widely. A 2008 survey by Association of American Colleges and Universities (AAC&U) found 137 (16%) of its 837 members reported offering majors, minors, or concentrations in public health (9). In his review of five college directories, Lee found overlapping but not identical listings for 154 U.S. baccalaureate degrees in public health, public health education, and public health nursing (10). A 2008 Internet search revealed more than 40 U.S. undergraduate programs in public health, community health, or health promotion (11). This variation in estimates called attention to how undergraduate programs are defined and counted.

The purpose of this study was to assess the number and characteristics of public health baccalaureate programs in the U.S. in 2013, and in turn, their growing popularity by comparing reported and demonstrated educational activity. The methodology mirrored the path taken by potential undergraduate students of selecting their programs of interest by referring to college directories and websites of universities.

## Methods

The nature and content of an undergraduate degree are typically captured by Bachelor of Arts (B.A.) or Bachelor of Science (B.S.) designations, as well as majors and minors. For the purposes of this analysis, an undergraduate degree in public health was defined as a major leading to a B.S., B.A., or other baccalaureate degree in public health or public health studies, which provides students with a strong general background in public health, or specialized training in at least one of the five core disciplines of public health: health policy and management, health behavior, biostatistics, epidemiology, and environmental health (12, 13).

The researchers reviewed three 2013 college directories widely used by high school students in selecting a university, and the Association of School and Programs in Public Health (ASPPH) listing of institutions offering undergraduate education in public health (14–17). The three directories and ASPPH offered overlapping but not identical listings. The initial review identified a combined inventory of 112 U.S. colleges and universities that used the phrase “public health” in their listings of baccalaureate degrees offered.

Fifty-eight institutions were subsequently excluded because they reported exclusively offering freestanding courses rather than awarding a public health degree (18) and offered a related major listed in another discipline. For example, health promotion as listed in kinesiology or environmental health is listed in engineering.

The degree title of each of the remaining 112 programs was then compared to its description on the institution’s website. Information on the website was used to assess curricula of programs, mission statements, required and elective coursework, field experience, and other characteristics to provide a foundation for baccalaureate programs. The Carnegie Commission on Higher Education classifications describing institutional diversity in U.S. higher education were applied to assess the characteristics of institutions offering baccalaureate degrees in public health (e.g., public vs. private; doctoral/research university, master’s college and university, or baccalaureate or associate college) (19, 20).

## Results

Upon examination, 54 of the original 112 institutions reporting a baccalaureate degree in public health matched the aforementioned definition of the undergraduate public health degree. Review of the institutions’ websites revealed notable variation in program characteristics and contents. The researchers created a template to summarize and interpret the findings.

Each of the 54 identified institutions offered a 4-year major. Upon successful completion of a 4-year program, most of the institutions award a B.S. degree in public health. Fewer award a B.A. or a B.S. in Public Health Studies. Less than one-fourth of schools offer a 5-year dual degree program, concurrently awarding a B.A. or B.S. degree and either a Master of Public Health (MPH) or Master of Health Sciences degree.

Most institutions provide their undergraduates with an opportunity to obtain general knowledge of public health combined with a specialization in a core area of public health. The most frequently offered specializations are health management and policy, environmental health, and health promotion and behavior. Health education (which includes both public health and community health education) is also identified as a public health specialization on the websites. Health informatics and epidemiology specializations are offered less frequently. While some institutions offer more than one specialization in the public health major, no institution offer specialization in all five of the core areas of public health.

As a general pattern in the 54 programs, undergraduate students are required to meet prerequisites in biological science, mathematics, psychology, and chemistry, as well as completing public health requirements (e.g., introductory courses in biostatistics, epidemiology, environmental health, health policy and management, and community health). Near the completion of their academic coursework, undergraduate students in most of the universities participate in a field practice experience.

The expectations for the baccalaureate graduates, as articulated by the programs’ information on their websites, are three-fold. First, students may pursue graduate or professional education. Programs with an emphasis in natural sciences prepare students for developing their post-graduate professional careers in areas including medicine and dentistry. Second, undergraduate students are expected to be prepared for entry-level public health practice positions in a variety of settings, including health departments, community agencies and organizations, schools, and clinical settings. Third, consistent with the Educated Citizen and Public Health model (vs. the existing professional model) (6), the undergraduate public health programs provide students with basic understanding of health concepts as they relate to behavioral, environmental, economic, housing, occupational, and social welfare issues.

Using the Carnegie Commission on Higher Education’s classification, the vast majority (over 60%) of the programs are located in the public institutions classified as “research institutions at the master’s and doctoral levels.” Unlike “baccalaureate colleges” that award undergraduate degrees exclusively, doctoral and master’s colleges and universities also offer graduate degrees across different disciplines.

## Discussion

Of the original 112 programs identified in the three college directories and the ASPPH database, the websites of only 54 of the 2,968 4-year institutions of higher education in the U.S. (21) provided information describing active undergraduate public health programs awarding baccalaureate degree in public health as defined for this study. This finding was surprising. Given the national attention to undergraduate public health education including ASPPH, AAC&U, and media such as *the Washington Post*, the researchers anticipated a larger number of programs, particularly in liberal arts colleges known to primarily offer baccalaureate degree programs.

The underlying reasons for the discrepancies between the number of public health programs reported in the directories and the actual number might be associated with the methods of the college directory publishers or the reporting by colleges and universities. The false positive reporting is also reflected in the discrepancies between the three directories. One might expect very similar, if not identical, reporting by all directories, but this was not the case. For example, 11 schools were listed in the *College Blue Book* (14), 54 schools were listed in the *Barron’s Profile* (15), and 70 schools were listed in *U.S. News College Compass* (16).

In addition to potential variation in their definitions of a public health degree, some schools might furnish information on proposed undergraduate programs. Public health degree programs that are not reported in the directories may also exist.

The study findings differ from those of the AAC&U and of the de Beaumont Foundation and ASPPH based upon both source and program definition. AAC&U’s 2008 Catalog Scan of Undergraduate Public Health Programs identified 93 institutions that offered a major in public health (9). The AAC&U data were gathered from online published materials of a specific subset of AAC&U’s 1,181 member institutions. Majors, minors, and concentrations in community health and other related fields were considered public health if they included the primary components of public health education for undergraduates (including courses in epidemiology, health systems, and others) (9); whereas this study applied a more traditional definition of an undergraduate degree in public health rather than a specialization.

Difference in operationalization of a public health degree used by the National Center for Education Statistics (NCES) may also explain why these study findings differed from other recently published reports. The de Beaumont Foundation and ASPPH relied on secondary analysis of data, specifically the Classification of Instruction Program (CIP) codes obtained from the Integrated Postsecondary Education Data System (IPEDS). Based on the analysis of the CIP codes, by 2012, 176 institutions awarded undergraduate public health degree. The CIP codes rely on self-reporting by university registrars of the number of awarded degrees, and more importantly, on the definition of IPEDS category of the degree. Although degrees in related fields (e.g., exercise and nutrition science) have their own CIP code in the NCES system, the degree may be misclassified as “public health.” The misclassification may be attributed to potential institutional differences in the interpretation of what constitutes a public health degree and as a result, in programs’ contents. In fact, a sensitivity analyses done by excluding institutions with fewer than 10 graduates per year found the number of institutions decreased by one-third. As noted by the researchers themselves, the decrease suggests that the number of institutions actually awarding public health degrees is smaller than alluded in the NCES data (2).

The study here used schools’ websites to validate the information provided by the directories. A direct follow-up with individual institutions might have changed the findings. This was beyond the scope of the current investigation, as the researchers were interested in availability of and ease of access to the information from the perspective of potential students.

In November 2006, the Consensus Conference on Undergraduate Public Health Education presented recommendations on curriculum frameworks and learning outcomes and methods for integrating those recommendations into the nation’s long-term strategy for public health education. It was noted that websites should be developed “to provide information on undergraduate public health and to share curriculum materials (22).” This recommendation remains vital. Colleges and universities should put extra emphasis on posting accurate and detailed information on availability and contents of undergraduate public health programs for prospective students.

In search of a program, potential students may inquire about its accreditation associated with a well-rounded education based upon the five core public health areas and with assurance that the education program in a school of their choice has met an agreed upon standard of quality (23, 24). Specialty accreditation of schools and programs is also available through the Council on Education for Public Health (CEPH). While CEPH accreditation was provided for undergraduate programs located in accredited schools of public health, undergraduate degrees associated with accredited programs in public health only became eligible for inclusion in the accreditation process in 2005 (25). Out of 49 schools and 99 programs accredited by CEPH in the U.S. (26), only 17 schools and 10 programs are included in our final listing of undergraduate programs. In September 2013, CEPH approved procedures to include “Standalone Baccalaureate Programs (SBPs)” as a unit of accreditation (27). Presently, there are no accredited SBPs in public health, and only 13 programs are presently applicants for review^1^ (28).

The finding that the majority of the programs were located in the public institutions classified by the Carnegie Commission as research institutions at the master’s and doctoral levels was unexpected. The majority of the programs were expected to be in institutions classified as baccalaureate, as these are the institutions that would be more likely to invest first in new undergraduate programs to attract students. Perhaps, the academic and financial burden associated with establishment of a new undergraduate program is easier for the institutions with preexisting public health programs at master’s and doctoral levels due to the availability of public health faculty and graduate teaching assistants.

## Conclusion

Based on the review of the three 2013 college directories and the ASPPH listing of institutions offering undergraduate education in public health (14–17), followed by the review of the programs’ websites, wide variation appeared in both the characteristics and assessment of the number of the undergraduate programs in public health. Variations in definition of what constitutes a baccalaureate degree in public health have implications for both the students seeking an undergraduate degree in public health, and the potential employers or graduate schools admitting these students. Discrepancies between college directories and information posted on programs’ websites may pose challenges to potential students and their families seeking to pursue undergraduate training toward a baccalaureate degree in public health.

The study could confirm that only 54 baccalaureate public health degrees are currently offered among the 2,968 institutions of higher learning in the U.S. (21). Due to surging demand for public health education, it is now on the agenda for many national organizations, schools and programs in public health, and their universities that will not wait for a national model to be finalized. As the *Washington Post* has reported, indeed, public health is a “hot field” when it comes to the interest that it generates. Despite the different findings of the various studies, there does appear to be noteworthy and rapid growth in undergraduate public health education, the question is one of magnitude and content.

## Conflict of Interest Statement

The authors declare that the research was conducted in the absence of any commercial or financial relationships that could be construed as a potential conflict of interest.

## Footnotes

^1^As of November 6, 2014, CEPH published 13 SBP applicants for accreditation. Six are included in the list of 54. Five programs offering either a Bachelor of Health Science, Applied Science, Science in Applied Health, or Science in Public Health were not listed in the directories. The remaining two programs did not meet the definition as their websites focused either on a specialization rather than a generalist public health focus or offered a degree in health promotion. No CEPH applicants were included in the ASPPH listing, and AAC&U listing included only four applicants. This variation reiterates the immaturity of undergraduate education.
